# Prevalence and associated factors of frailty in hospitalized elderly patients with heart failure

**DOI:** 10.3389/fcvm.2026.1902415

**Published:** 2026-09-14

**Authors:** Xiu-Fang Hong, Ke-Xin Shen, Fang-Ying Li, Hui-Lan Guan, Jing Yan

**Affiliations:** 1 Department of Geriatrics, Affiliated Zhejiang Hospital, Zhejiang University School of Medicine, Hangzhou, China; 2 Zhejiang University School of Medicine, Hangzhou, China

**Keywords:** comprehensive geriatric assessment, elderly, frailty, heart failure, nutritional status, physical function

## Abstract

**Background:**

Frailty is frequently observed in elderly hospitalized patients with heart failure. Recognizing its prevalence and associated factors is essential for early identification and may help reduce socioeconomic burdens. This study aimed to assess the prevalence, clinical features, and correlates of frailty in this population.

**Methods:**

This single-center cross-sectional study enrolled consecutive hospitalized patients aged≥60 years with heart failure from January 2023 to December 2023. According to the Clinical Frailty Scale (CFS), patients were classified into three groups: non-frailty (CFS 1–4), mild-to-moderate frailty (CFS 5–6), and severe frailty (CFS 7). Clinical characteristics were compared across groups using comprehensive geriatric assessment data and auxiliary examination results. A multivariable partial proportional odds model (PPOM) was employed to identify factors associated with frailty severity.

**Results:**

A total of 298 elderly patients with heart failure were enrolled, including 149 males (50.0%). The age of the subjects ranged from 60 to 101 years, with a median of 83 years. Amongst them, 234 (78.5%) were identified as having frailty, of whom 192 (82.1%) presented with mild-to-moderate frailty. Statistically significant differences were observed among the three groups in age, weight, body mass index, comorbidity status, activities of daily living, instrumental activities of daily living, mini-nutritional assessment short-form, short physical performance battery, performance-oriented mobility assessment, dominant calf circumference, grip strength, B-type natriuretic peptide, left ventricular ejection fraction (all *P* < 0.05). No statistically significant differences were found among the three groups in sex, height, hypertension, diabetes, coronary artery disease, arrhythmia, malnutrition, electrocardiographic abnormality grade (including atrial fibrillation, high voltage, T-wave changes, and ST-segment changes), or echocardiographic abnormality grade (including left heart enlargement and pulmonary hypertension) (all *P* > 0.05). Multivariate PPOM analysis indicated that comorbidity status, mini-nutritional assessment short-form, short physical performance battery, and left ventricular ejection fraction were significant associated factors for frailty in elderly patients with heart failure (all *P* < 0.05).

**Conclusions:**

Hospitalized patients with heart failure show a high prevalence of frailty. Comorbidities, poor nutritional status (lower MNA-SF score), impaired physical function (lower SPPB score), and reduced LVEF are important correlates of frailty severity in these patients.

## 1 Introduction

With steady improvements in healthcare and socioeconomic development, global life expectancy has increased, and aging is accelerating both domestically and internationally. Consequently, age-related diseases have become a major public health concern. Heart failure (HF), a clinical syndrome caused by structural or functional cardiac disorders leading to insufficient cardiac output (1), is the end stage of various cardiovascular diseases. It predominantly affects the elderly and is characterized by high incidence, high mortality, high hospitalization rates, poor quality of life, and high medical costs (1). Frailty is a core phenotype of geriatric syndromes, characterized by increased vulnerability and reduced stress resistance in elderly patients, where even minor stressors can trigger negative clinical events (2). As indicated by previous findings, frailty is a significant comorbidity that negatively impacts the prognosis of HF in the elderly, serving as a reliable factor of short-term mortality, readmission, and disability in HF patients (3–5). Conversely, HF itself is also closely associated with the development of frailty (6). The coexistence of these conditions may create a complex interplay, which could contribute to a mutually detrimental process, reduce quality of life, and place a heavy burden on healthcare systems. Research on HF combined with frailty in elderly patients is still developing in China. This study aims to investigate the prevalence of different frailty degrees and their associated factors in elderly HF patients, and lay a theoretical foundation for exploring comprehensive geriatric assessment (CGA)-based approaches for identifying high-risk patients.

## 2 Materials and methods

### 2.1 Study patients

The research was a single-center, cross-sectional study with retrospective data collection. The sample consisted of consecutive hospitalized elderly patients with HF aged ≥60 years in the internal medicine wards of Zhejiang Hospital, Zhejiang University School of Medicine, from January 2023 to December 2023. A total of 298 patients were included in the final analysis (Figure 1).

**Figure 1:**
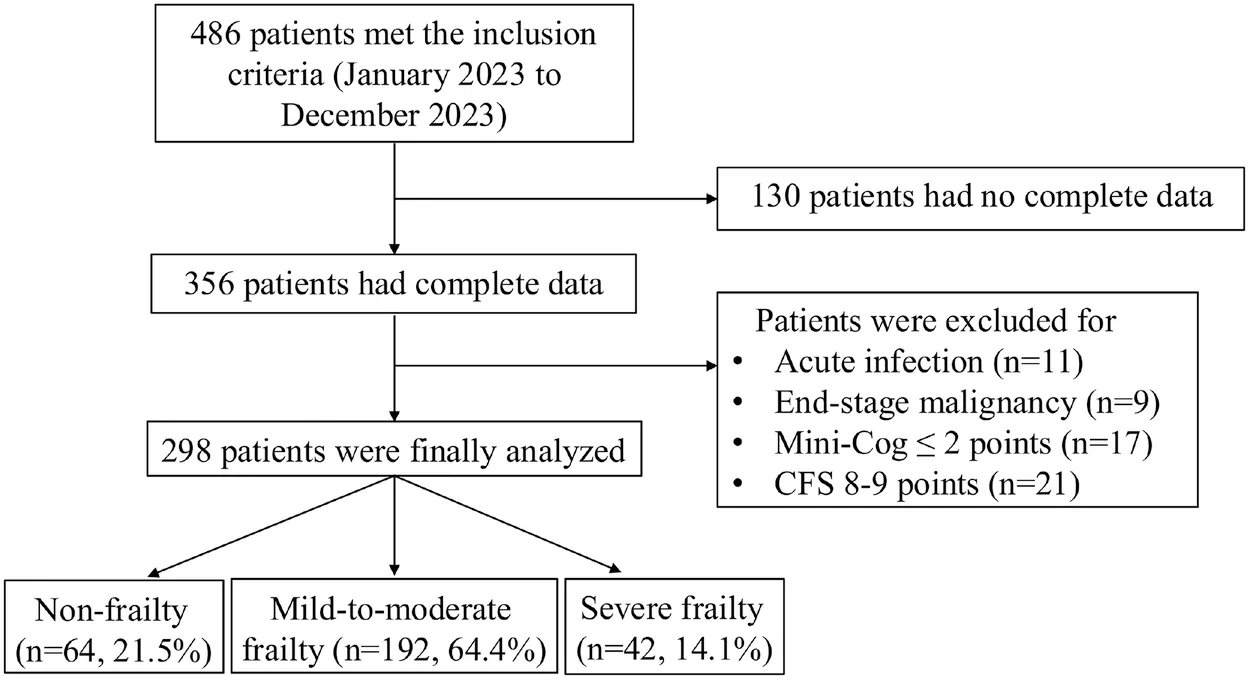
Flowchart of the patient inclusion.

### 2.2 Inclusion and exclusion criteria

Inclusion criteria: (1) age ≥60 years; (2) completion of CGA, B-type natriuretic peptide (BNP), electrocardiography (ECG), and echocardiography (Echo); (3) complete data and clinical diagnosis consistent with HF (i.e., presence of typical symptoms and signs, accompanied by Echo confirmed cardiac structural and/or functional abnormalities, and/or elevated BNP levels) (7).

Exclusion criteria: (1) incomplete data; (2) acute infection; (3) end-stage malignancy; (4) significant cognitive impairment (Mini-Cognitive Assessment Instrument [Mini-Cog] total score ≤2 points) (8); (5) very severe frailty or terminal illness (Clinical Frailty Scale [CFS] score of 8–9 points) (9).

All data were anonymized, and the study was approved by the hospital ethics committee (ZJHIRB-2026-036K).

### 2.3 Study methods

#### 2.3.1 Data collection

Data were sourced from the CGA database and the medical record database of Zhejiang Hospital, Zhejiang University School of Medicine. Hospitalized cases meeting HF diagnostic criteria and completing geriatric assessment were selected. Baseline information (age, sex, height, weight with calculated BMI, comorbidity status, hypertension, diabetes, coronary artery disease, arrhythmia, and documented malnutrition), frailty assessment results, and scores from CGA scales, including activities of daily living (ADL), instrumental activities of daily living (IADL), Mini-Nutritional Assessment Short-Form (MNA-SF), Short Physical Performance Battery (SPPB), and Performance-Oriented Mobility Assessment (POMA), were recorded. All assessments were performed within the first three days of hospitalization by qualified geriatric nurses trained in standardized CGA. Plasma BNP was measured in our hospital's central clinical laboratory using a unified detection platform and reagent kit. LVEF was assessed via transthoracic echocardiography, conducted by qualified sonographers according to the standard local echocardiography protocol. All measurements were completed within our single study centre with consistent operational standards.

#### 2.3.2 Frailty assessment

The Clinical Frailty Scale (CFS) is a useful tool for assessing frailty in elderly cardiovascular patients, with good sensitivity and specificity. CFS grades patients into 9 levels: 1 = very fit, 2 = fit, 3 = managing well, 4 = vulnerable, 5 = mildly frail, 6 = moderately frail, 7 = severely frail, 8 = very severely frail, 9 = terminally ill. Higher scores indicate greater frailty. Patients with scores 8–9 were excluded because these categories represent very severe frailty and terminal illness, respectively, in which profound functional impairment or critical clinical status precludes the completion of a comprehensive geriatric assessment (CGA). Specifically, patients at these levels are typically unable to perform physical function tests (e.g., SPPB, POMA, handgrip strength) or cooperate with nutritional and cognitive assessments, which would result in substantial missing data and compromise the validity of the multivariate regression analysis. Therefore, to ensure data completeness and the reliability of CGA-based correlates, patients with CFS 8–9 were not included in the present analysis. Scores 1-4 were classified as non-frailty, 5-6 as mild-to-moderate frailty, and 7 as severe frailty (9).

#### 2.3.3 Physical function assessment

##### 2.3.3.1 Activities of daily living

ADL and IADL were assessed through direct observation or indirect questioning, with higher scores reflecting greater independence. ADL was evaluated using the Barthel Index and categorized as follows: high level (100 points, complete independence), moderate level (61–99 points), and low level (≤60 points, significant impairment) (10). IADL was assessed using the Lawton Scale, with scores classified as high level (7–8 points), moderate level (5–6 points), and low level (≤4 points) (11).

##### 2.3.3.2 Motor function assessment

The POMA and the SPPB are commonly used scales for assessing fall risk in older adults. The POMA has good reliability and validity for balance and gait assessment (12), with scores ranging from 0 to 28 and categorized as: ≥24 high function, 18–23 moderate function, and ≤17 low function (lower scores indicate worse performance and higher fall risk) (13). The SPPB assesses lower extremity function through balance, gait speed, and chair stand performance, with scores 0–12: 8–12 normal function, 4–7 moderate impairment, and 0–3 severe impairment (14). In addition, grip strength was measured using a Jamar dynamometer (kg), with sex-specific cutoffs for weakness (<28 kg for males, <18 kg for females) (15).

#### 2.3.4 Nutritional status assessment

The MNA-SF was used to assess nutritional status (16). The scale consists of six items (e.g., weight change, food intake, limb measurement) and yields a total score of 0–14, with lower scores indicating a higher risk of malnutrition. Scores ≤7 are classified as malnourished, 8–11 as at risk, and 12–14 as well-nourished (16). Additionally, dominant calf circumference (cm) was recorded, and sex-specific thresholds for low muscle mass were applied (<34 cm for males, <33 cm for females) (15).

#### 2.3.5 Auxiliary examinations

Data on BNP, ECG, and echocardiography (including left ventricular ejection fraction, LVEF) were collected from medical records of patients with a confirmed diagnosis of HF during hospitalization. The normal clinical range for BNP is <100 ng/L, whereas a value ≥100 ng/L indicates acute heart failure or acute exacerbation of chronic HF (7). In this study, LVEF ≤50% was defined as reduced ejection fraction (17). To better characterize cardiac abnormality severity for baseline inter-group comparisons, this study exploratorily designed two composite scoring systems. The ECG abnormality score (0–4) assigned 1 point each for atrial fibrillation, high voltage, T-wave changes, and ST-segment changes. The Echo abnormality grade (0–5) assigned 1 point each for left heart enlargement, moderate or severe valvular regurgitation, pulmonary hypertension, left ventricular diastolic dysfunction and reduced ejection fraction. Given the limitation that these self-developed scores lacked external validation, they were employed solely for baseline intergroup comparisons.

### 2.4 Statistical analysis

SPSS 26.0 and Stata 16.0 were used for statistical analysis. The normally distributed continuous data were presented as mean and SDs, and the nonnormally distributed continuous data were presented as medians and IQR. Categorical variables were presented as percentages or constituent ratios. First, overall comparisons across the three frailty groups were performed using one-way ANOVA test (normally distributed continuous data), Pearson's chi-squared test (categorical data), and Kruskal–Wallis test (non-normally distributed continuous data). Then, *post-hoc* pairwise comparisons were only conducted if the overall group comparison reached *P* < 0.05. For normally-distributed variables with significant overall differences, the LSD *t*-test was used for pairwise comparisons; for non-normally distributed variables, the Mann–Whitney *U*-test was applied for *post-hoc* testing.

Univariate ordinal logistic regression was performed for each variable with a *P* *<* 0.05 in the intergroup comparisons, and the odds ratio (OR) with its 95% confidence interval (CI) was calculated. To avoid multicollinearity, Spearman's correlation analysis was first applied to candidate variables within the same clinical domain (e.g., physical function, nutritional status). When the absolute correlation coefficient (∣*r_s_*∣) exceeded 0.6, only the variable with greater clinical relevance was retained. Subsequently, variance inflation factor (VIF) analysis was performed for all variables retained in the final model, with VIF <5 considered to indicate no significant multicollinearity (18).

With the ordinal three-level frailty grade as the dependent variable, the selected independent variables were entered into the final model. The proportional odds assumption was first assessed using the test of parallel lines. If satisfied (*P* ≥ 0.05), multivariable proportional odds ordinal logistic regression was used in SPSS, with all variables reported as common odds ratios. If violated (*P* < 0.05), a multivariable partial proportional odds ordinal logistic regression model (PPOM) was fitted using Stata's gologit2 program with the autofit option, which reports proportional-odds effects as common odds ratios and partial effects as cutpoint-specific odds ratios (19).

In addition, a sensitivity analysis was performed by refitting the final model with continuous specifications of the main factors to examine the robustness of categorical cutpoint choices. *P* *<* 0.05 was considered statistically significant.

## 3 Results

### 3.1 General clinical data

A total of 298 elderly hospitalized HF patients with complete data were included, 149 males and 149 females, median age 83 years (range 60–101). Non-frailty was present in 64 patients (21.5%), mild-to-moderate frailty in 192 (64.4%), and severe frailty in 42 (14.1%). Over half (64.1%) had comorbidities, 28.5% had diabetes, 75.8% had hypertension, 20.5% had coronary heart disease, 25.2% had arrhythmias (including atrial fibrillation, ventricular/atrial premature beats), and 3.0% had malnutrition or hypoalbuminemia.

### 3.2 Clinical characteristics and frailty severity differences in hospitalized elderly HF patients

Compared with non-frail patients, frail patients tended to be older, had lower body weight, and had a lower BMI (all *P* < 0.05). The median age in the non-frailty group was 75.00 years, which was lower than that in the mild-to-moderate frailty group (85.00 years) and the severe frailty group (85.00 years) (all *P* < 0.001). Body weight and BMI in the non-frailty group (62.37 ± 11.18 kg, 23.31 ± 3.17 kg/m^2^) were significantly higher than those in the mild-to-moderate frailty group (56.17 ± 10.79 kg, 21.75 ± 4.01 kg/m^2^) and the severe frailty group (56.15 ± 10.09 kg, 21.37 ± 3.38 kg/m^2^) (*P* < 0.05), whereas no statistically significant difference was observed between the mild-to-moderate and severe frailty groups (*P* > 0.05). The presence of comorbidity was significantly higher in the frailty groups (mild-to-moderate frailty: 68.2%; severe frailty: 71.4%) than in the non-frailty group (46.9%) (all adjusted *P* < 0.05). No statistically significant differences were found among the three groups in sex, height, or the prevalence of hypertension, diabetes, coronary artery disease, arrhythmia, or malnutrition (all *P* > 0.05).

Frail patients exhibited a marked lower level of physical function, which was inversely associated with frailty severity. ADL scores decreased stepwise from 100 in the non-frailty group to 85 in the mild-to-moderate frailty group and 25 in the severe frailty group (all adjusted *P* < 0.001). Similarly, IADL scores (8, 3, and 0, respectively), SPPB scores (10, 4, and 0), POMA scores (27, 17, and 0), and grip strength (25.20 kg, 16.50 kg, and 9.85 kg) differed significantly across the three groups (all adjusted *P* < 0.001). Nutritional status was also poorer in patients with greater frailty severity. MNA-SF scores in the non-frailty, mild-to-moderate frailty, and severe frailty groups were 12, 9, and 6, respectively (all adjusted *P* < 0.001), and dominant calf circumference decreased correspondingly from 33.5 cm to 31 cm and 29.2 cm (adjusted *P* < 0.001).

BNP levels differed significantly across the three groups (adjusted *P* < 0.001). Frail patients with heart failure (mild-to-moderate frailty: 142.50 ng/L; severe frailty: 199.50 ng/L) had higher BNP levels than the non-frailty group (70.00 ng/L) (adjusted *P* < 0.001 for both comparisons), whereas no significant difference was observed between the mild-to-moderate and severe frailty groups (adjusted *P* = 0.949). Additionally, the proportion of patients with reduced ejection fraction was progressively higher across frailty severity (3.1%, 15.6%, and 33.3%, respectively) and was significantly higher in the severe frailty group than in the other two groups (adjusted *P* < 0.05). In contrast, no significant differences were observed among the three groups in neither the ECG abnormality grade or its components (including atrial fibrillation, high voltage, T-wave changes, and ST-segment changes), nor in the echocardiographic abnormality grade or its components (including left heart enlargement, pulmonary hypertension, moderate-to-severe valvular regurgitation, and left ventricular diastolic dysfunction) (all adjusted *P* > 0.05). Details are presented in Table 1.

**Table 1:** Comparison of clinical data, CGA indices, and auxiliary examination results among elderly hospitalized HF patients.

Variables	Total (*n* = 298)	Non-frailty (*n* = 64, 21.5%)	Mild-to-moderate frailty (*n* = 192, 64.4%)	Severe frailty (*n* = 42, 14.1%)	*P*-value
Baseline Characteristics					
Age (years)	83.00 (75.00–88.00)	75.00 (69.25–81.00)	85.00 (77.00–89.00)*	85.00 (77.00–89.00)**	<0.001^c^
Sex, male [n (%)]	149 (50.0)	30 (46.9)	99 (51.6)	20 (47.6)	0.766^b^
Height (cm)	161.23 ± 8.67	161.94 ± 8.58	160.83 ± 8.91	161.98 ± 7.69	0.569^a^
Weight (kg)	57.31 ± 10.96	62.37 ± 11.18	56.17 ± 10.79*	56.15 ± 10.09**	0.003^a^
BMI (kg/m²)	22.04 ± 3.81	23.31 ± 3.17	21.75 ± 4.01*	21.37 ± 3.38**	0.008^a^
Comorbidity [n (%)]	191 (64.1)	30 (46.9)	131 (68.2)*	30 (71.4)**	0.005^b^
Hypertension [n (%)]	226 (75.8)	48 (75.0)	145 (75.5)	33 (78.6)	0.902^b^
Diabetes mellitus [n (%)]	85 (28.5)	12 (18.8)	58 (30.2)	15 (35.7)	0.115^b^
Coronary heart disease [n (%)]	61 (20.5)	16 (25.0)	38 (19.8)	7 (16.7)	0.540^b^
Arrhythmia [n (%)]	75 (25.2)	17 (26.6)	50 (26.0)	8 (19.0)	0.613^b^
Malnutrition [n (%)]	9 (3.0)	1 (1.6)	6 (3.1)	2 (4.8)	0.636^b^
CGA Results					
ADL score (points)	90.00 (60.00–100.00)	100.00 (100.00–100.00)	85.00 (65.00–95.00)*	25.00 (13.75–40.00)**^,^***	<0.001^c^
IADL score (points)	3.00 (1.00–6.00)	8.00 (8.00–8.00)	3.00 (1.00–5.00)*	0.00 (0.00–1.00)**^,^***	<0.001^c^
MNA-SF score (points)	10.00 (7.00–12.00)	12.00 (11.00–14.00)	9.00 (7.00–12.00)*	6.00 (4.00–9.00)**^,^***	<0.001^c^
SPPB score (points)	3.00 (1.00–6.00)	10.00 (8.00–11.50)	4.00 (1.00–6.00)*	0.00 (0.00–0.00)**^,^***	<0.001^c^
POMA score (points)	18.0 (1.0–26.0)	27.0 (26.0–28.0)	17.0 (6.5–23.0)*	0.00 (0.00–0.00)**^,^***	<0.001^c^
Dominant calf circumference (cm)	31.50 (28.50–34.00)	33.50 (31.90–36.00)	31.00 (28.50–33.00)*	29.20 (25.75–31.45)**^,^***	<0.001^c^
Grip strength (kg)	17.50 (11.88–23.93)	25.20 (18.80–31.10)	16.50 (11.90–22.10)*	9.85 (5.58–14.35)**	<0.001^c^
Cardiac Findings					
BNP (ng/L)	135.00 (51.75–275.75)	70.00 (24.25–164.75)	142.50 (70.00–283.25)*	199.50 (75.50–401.25)**	<0.001^c^
ECG abnormality grade (points)	1.0 (0.0–1.0)	1.0 (0.0–1.0)	1.0 (0.0–1.0)	1.0 (0.0–1.0)	0.276^c^
Atrial fibrillation [n (%)]	49 (16.4)	10 (15.6)	30 (15.6)	9 (21.4)	0.643^b^
High voltage [n (%)]	29 (9.7)	6 (9.4)	15 (7.8)	8 (19.0)	0.084^b^
T-wave change [n (%)]	57 (19.1)	8 (12.5)	41 (21.4)	8 (19.0)	0.296^b^
ST-segment change [n (%)]	80 (26.8)	21 (32.8)	45 (23.4)	14 (33.3)	0.202^b^
Echo abnormality grade (points)	1.0 (1.0–2.0)	1.0 (1.0–2.0)	1.0 (1.0–2.0)	2.0 (1.0–2.0)	0.447^c^
Reduced ejection fraction [n (%)]	46 (15.4)	2 (3.1)	30 (15.6)*	14 (33.3)**^,^***	<0.001^b^
Left heart enlargement [n (%)]	204 (68.5)	43 (67.2)	132 (68.8)	29 (69.0)	0.889^b^
Pulmonary hypertension [n (%)]	45 (15.1)	6 (9.4)	32 (16.7)	7 (16.7)	0.324^b^
Moderate/severe valvular regurgitation [n (%)]	50 (16.8)	11 (17.2)	36 (18.8)	3 (7.1)	0.189^b^
Left ventricular diastolic dysfunction [n (%)]	102 (34.2)	25 (39.1)	69 (35.9)	8 (19.0)	0.081^b^
LVEF (%)	63.21 ± 9.67	65.37 ± 6.87	63.11 ± 10.27	60.35 ± 9.92**	0.034^a^

Omnibus three-group comparisons:

a one-way ANOVA for normally distributed continuous data.

b Pearson's chi-squared test for categorical data.

c Kruskal–Wallis test for non-normally distributed continuous data. Post-hoc pairwise comparisons were only performed when the omnibus three-group test yielded *P* < 0.05. For pairwise comparisons: LSD *t*-test for normally distributed continuous data; chi-squared test for categorical data; Mann–Whitney *U*-test for non-normally distributed continuous data.

* *P* *<* 0.05 vs. non-frailty.

** *P* *<* 0.05 vs. mild-to-moderate frailty.

*** *P* *<* 0.05 vs. non-frailty.

ECG abnormality grade (0–4 points): 1 point each for atrial fibrillation, high voltage, T-wave changes, and ST-segment changes; Echo abnormality grade (0–5 points): 1 point each for left heart enlargement, moderate or severe valvular regurgitation, pulmonary hypertension, left ventricular diastolic dysfunction and reduced ejection fraction.

BMI, body mass index; ADL, activities of daily living; IADL, instrumental ADL; GDS, Geriatric Depression Scale; MMSE, Mini-Mental State Examination; MNA-SF, mini nutritional assessment-short form; SPPB, Short Physical Performance Battery; POMA, performance-oriented mobility assessment; SMI, skeletal muscle mass index; BNP, B-type Natriuretic Peptide; LVEF, Left Ventricular Ejection Fraction.

### 3.3 Univariate ordinal logistic regression analysis of hospitalized elderly HF patients with frailty

Univariate ordinal logistic regression analysis was performed with frailty grade (non-frailty, mild-to-moderate frailty, severe frailty) as the ordinal dependent variable. Each variable with *P* < 0.05 in the intergroup comparisons (Table 1) was entered into the model, and the OR with its 95% confidence interval was calculated. The results showed that age, comorbidity, and BNP level were positively associated with frailty grade (all *P* < 0.05), whereas body weight, BMI, ADL, IADL, MNA-SF, SPPB, POMA, dominant calf circumference, handgrip strength, and LVEF level were negatively associated with frailty grade (all *P* < 0.05). Details are presented in Table 2.

**Table 2:** Univariate ordinal logistic regression analysis of factors associated with frailty in hospitalized older HF patients.

Factors	B (Estimate)	Standard error	Wald	*P*_-value_	OR	95% CI
Age	0.133	0.021	40.595	<0.001	1.142	1.096–1.190
Weight	−0.043	0.013	10.780	0.001	0.958	0.934–0.983
BMI	−0.112	0.038	8.781	0.003	0.894	0.830–0.963
Comorbidity	0.916	0.287	10.154	0.001	2.500	1.423–4.391
ADL	−0.186	0.039	23.374	<0.001	0.830	0.770–0.895
IADL	−1.634	0.234	48.517	<0.001	0.195	0.123–0.309
MNA-SF	−0.447	0.070	40.840	<0.001	0.639	0.558–0.733
SPPB	−0.567	0.073	61.027	<0.001	0.567	0.492–0.654
POMA	−0.258	0.043	36.017	<0.001	0.773	0.711–0.841
Dominant calf circumference	−0.250	0.047	28.522	<0.001	0.779	0.711–0.854
Grip strength	−0.110	0.018	36.617	<0.001	0.896	0.864–0.928
BNP	0.004	0.001	11.233	0.001	1.004	1.002–1.006
LVEF	−3.284	1.653	3.948	0.047	0.037	0.001–0.956

Each associated factor was analyzed using the univariate logistic regression analysis. Frailty grade (non-frailty, mild-to-moderate frailty, severe frailty) was used as the ordinal dependent variable.

BMI, body mass index; MNA-SF, mini nutritional assessment-short form; SPPB, Short Physical Performance Battery; BNP, B-type Natriuretic Peptide; LVEF, Left Ventricular Ejection Fraction.

### 3.4 Independent variable selection and transformation for multivariable ordinal logistic regression

To avoid multicollinearity that could compromise the stability of the multivariable ordinal logistic regression results, collinearity screening and selection were performed among variables with *P* < 0.05 in the univariate analysis. For highly correlated indicator groups within the two domains of “physical function” (including ADL, IADL, SPPB, POMA, and grip strength) and “nutritional assessment” (including BMI, MNA-SF, body weight, and dominant calf circumference), Spearman correlation analysis was applied using a cutoff of ∣*r_s_*∣ > 0.6 to define high collinearity. From each domain, when collinearity was detected, only the indicator with the greatest clinical relevance was retained for entry into the multivariable model. Post-hoc VIF analysis confirmed the absence of severe multicollinearity among the retained variables.

#### 3.4.1 Physical function

SPPB, POMA, grip strength, ADL, and IADL are all associated with physical function. Spearman correlation analysis showed that SPPB was significantly correlated with grip strength (*r_s_* = 0.605), POMA (*r_s_* = 0.913), ADL (*r_s_* = 0.850), and IADL (*r_s_* = 0.775) (all *P* < 0.001) (Table 3). All correlation coefficients exceeded the threshold of |*r_s_*| > 0.6, indicating potential multicollinearity among these variables. Although ADL and IADL assess daily living abilities, POMA evaluates mobility, and grip strength reflects upper-extremity muscle performance, SPPB was selected as the representative indicator for the physical function domain because it specifically focuses on pure physical performance, comprehensively capturing standing balance, gait speed, and lower-limb strength. Furthermore, as a classic scale for frailty prediction, SPPB holds significant clinical value for early identification of frailty and assessment of the degree of physical decline. Therefore, SPPB was entered into the multivariable ordinal logistic regression.

**Table 3:** Spearman correlation analysis of indicators within the physical function domain.

Variable	ADL	IADL	SPPB	POMA	Grip strength
ADL	—	**0.838**	**0**.**850**	**0**.**865**	**0**.**602**
IADL	**0**.**838**	—	**0**.**775**	**0**.**777**	0.582
SPPB	**0**.**850**	**0**.**775**	—	**0**.**913**	**0**.**605**
POMA	**0**.**865**	**0**.**777**	**0**.**913**	—	**0**.**641**
Grip strength	**0**.**602**	0.582	**0**.**605**	**0**.**641**	—

All correlations were statistically significant at *P* < 0.01. Coefficients with |rₛ| > 0.6 are shown in bold, indicating potential multicollinearity.

#### 3.4.2 Nutritional status

Body weight, BMI, dominant calf circumference, and MNA-SF are all indicators of nutritional status. Spearman's rank correlation coefficients were calculated to assess the relationships among these variables. MNA-SF (all *P* < 0.001) was significantly correlated with Body weight (*r_s_* = 0.572), BMI (*r_s_* = 0.675), and dominant calf circumference (*r_s_* = 0.686) (Table 4). The absolute correlation coefficient between dominant calf circumference and MNA-SF exceeded 0.6 (|*r_s_*| > 0.6), indicating high collinearity, but dominant calf circumference's reliability as a single anthropometric measure may be compromised by frequent lower-limb oedema in hospitalized heart failure patients. Although the correlation coefficients of body weight with MNA-SF did not reach this threshold (|*r_s_* | < 0.6), MNA-SF was considered to provide a more comprehensive assessment of nutritional status in hospitalized elderly patients with heart failure, as it incorporates multidimensional information including global nutritional assessment, dietary intake, and body mass changes. Moreover, it is suitable for bedridden or mobility-limited individuals and has demonstrated both practicality and validity (20). Accordingly, the MNA-SF score was selected as the representative indicator for the nutritional domain and entered into the multivariable ordinal logistic regression.

**Table 4:** Spearman correlation analysis of indicators within the nutritional assessment domain.

Variable	BMI	Weight	MNA-SF	Dominant calf circumference
BMI	—	**0.829**	**0**.**675**	**0**.**721**
Weight	**0**.**829**	—	0.572	**0**.**759**
MNA-SF	**0**.**675**	0.572	—	**0**.**686**
Dominant calf circumference	**0**.**721**	**0**.**759**	**0**.**686**	—

All correlations were statistically significant at *P* < 0.01. Coefficients with |rₛ| > 0.6 are shown in bold, indicating potential multicollinearity.

#### 3.4.3 Variable transformation

To enhance the clinical applicability of the regression results, the continuous variables retained in the final model were transformed into categorical variables. In routine clinical practice, effect estimates corresponding to unit increments in continuous variables often fail to directly inform clinical decisions. In contrast, categorization using clinically meaningful cutoff points makes changes in individual risk strata more readily identifiable and actionable, thereby facilitating clinical practice. Moreover, such transformation enables meaningful comparisons with previous studies. Based on these considerations and with reference to prior literature, age (21), MNA-SF (22), SPPB (23), BNP (7), and LVEF (17) were converted into categorical variables according to previous research and clinically established cutoff points. Details are presented in Table 5.

**Table 5:** Assignment and categorization of continuous variables.

Variable	Assignment
Age	1 = 60–74 years, 2 = 75–84 years, 3=≥85 years
SPPB score	1 = 0–3 points, 2 = 4–7 points, 3=≥8 points
MNA-SF score	1 = 0–7 points, 2 = 8–11 points, 3=≥12 points
BNP	1 = BNP<100 ng/L, 2 = BNP≥100 ng/L
LVEF	1 = LVEF≤50%, 2 = LVEF>50%

#### 3.4.4 Collinearity diagnostics (VIF analysis)

To verify whether the final model suffered from multicollinearity, VIF analysis was performed for all independent variables retained in the multivariable ordinal logistic regression. As shown in Table 6, VIF values ranged from 1.054 to 1.556, all well below 5, indicating that no significant multicollinearity existed among the independent variables in the final model, and the model estimates were stable and reliable. Details are presented in Table 6.

**Table 6:** Variance inflation factor analysis for the final multivariable ordinal logistic regression model.

Variable	VIF	Tolerance (1/VIF)
Age	1.220	0.820
Comorbidity	1.054	0.949
SPPB	1.556	0.643
MNA-SF	1.391	0.719
BNP	1.177	0.850
LVEF	1.206	0.829

All variables were entered as categorical variables. All VIF values were <5, indicating no significant multicollinearity among the independent variables.

### 3.5 Partial proportional odds model analysis of factors associated with frailty in hospitalized elderly HF patients

The test of parallel lines yielded a significant result (*χ*^2^ = 171.024, df = 9, *P* < 0.001), indicating a violation of the proportional odds assumption. To address this, a multivariable PPOM was employed, with age, comorbidity, MNA-SF, SPPB, BNP and LVEF as categorical independent variables and frailty grade as the dependent variable. The results showed that comorbidity, MNA-SF, SPPB, and lower LVEF were significant factors associated with frailty status in hospitalized elderly patients with HF (all *P* < 0.05), while BNP showed no significant association (*P* = 0.512). Notably, age and the at-risk-of-malnutrition group (MNA-SF 8–11 points) exhibited threshold-specific effects, primarily distinguishing non-frailty from frailty (Mild-to-moderate frailty + Severe frailty). Details are presented in Figure 2.

**Figure 2:**
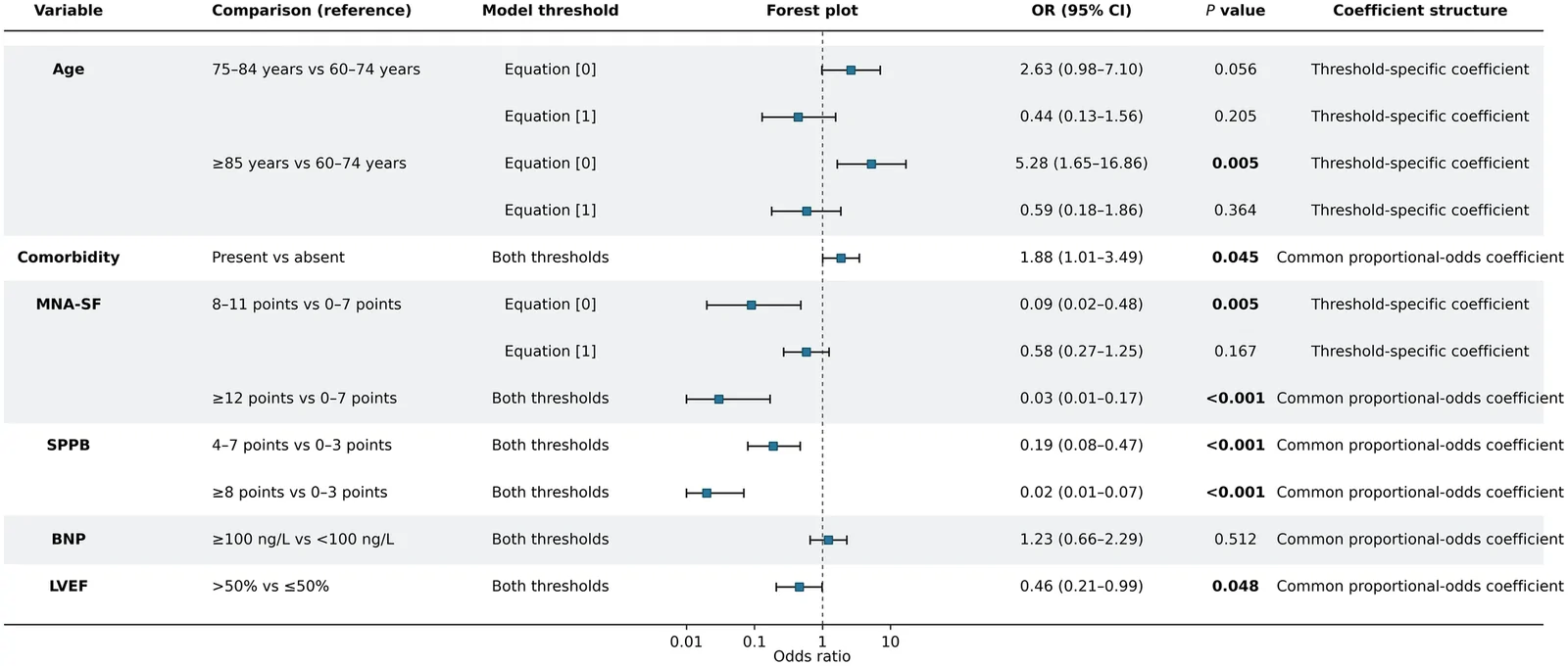
Primary analysis: multivariable PPOM analysis of factors associated with frailty in hospitalized elderly HF patients. Notes: Multivariable PPOM analysis was performed with frailty grade as the ordinal dependent variable. Equation [0]: Severe frailty plus mild-to-moderate frailty vs non-frailty; Equation [1]: Severe frailty vs non-frailty plus mild-to-moderate frailty. Adjusted odds ratios were mutually adjusted for all other variables shown in the figure. When the proportional-odds assumption was not satisfied, threshold-specific coefficients were estimated separately for Equation [0] and Equation [1]. When the proportional-odds assumption was satisfied, a common proportional-odds coefficient was estimated jointly for Equation [0] and Equation [1]. Squares represent aORs, horizontal lines represent 95% CIs, and the dashed vertical line indicates an aOR of 1.00. The forest-plot axis is displayed on a logarithmic scale. *P* <0.05 are shown in bold. Abbreviations: OR, odds ratio; CI, confidence interval; MNA-SF, Mini-Nutritional Assessment Short-Form; SPPB, Short Physical Performance Battery; BNP, B-type Natriuretic Peptide; LVEF, Left Ventricular Ejection Fraction.

### 3.6 Sensitivity analysis

Consistent with the categorical primary analysis (Figure 2), MNA-SF and SPPB remained significantly associated with frailty severity when entered as continuous variables (both *P* < 0.001). Age retained statistical significance in Equation 0 (non-frailty vs. frailty) (OR = 1.067, *P* = 0.024), whereas BNP remained non-significant (*P* > 0.05). Although comorbidity and LVEF did not reach statistical significance in the sensitivity analysis (*P* > 0.05), its effect direction remained consistent with that observed in the primary model. Details are presented in Figure 3.

**Figure 3:**
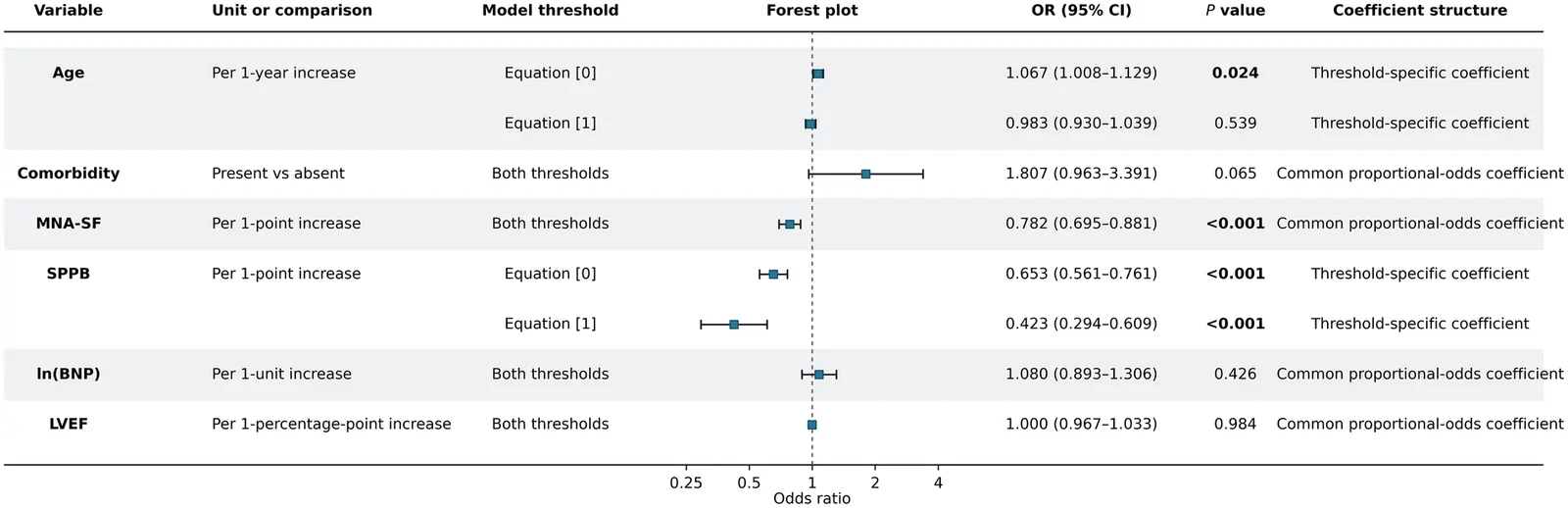
Sensitivity analysis: continuous-variable PPOM analysis of factors associated with frailty in hospitalized elderly HF patients. Notes: Multivariable PPOM analysis was performed with frailty grade as the ordinal dependent variable. Equation [0]: Severe frailty plus mild-to-moderate frailty vs non-frailty; Equation [1]: Severe frailty vs non-frailty plus mild-to-moderate frailty. Adjusted odds ratios were mutually adjusted for all other variables shown in the figure. When the proportional-odds assumption was not satisfied, threshold-specific coefficients were estimated separately for Equation [0] and Equation [1]. When the proportional-odds assumption was satisfied, a common proportional-odds coefficient was estimated jointly for Equation [0] and Equation [1]. Squares represent aORs, horizontal lines represent 95% CIs, and the dashed vertical line indicates an aOR of 1.00. The forest-plot axis is displayed on a logarithmic scale. *P* <0.05 are shown in bold. Abbreviations: OR, odds ratio; CI, confidence interval; MNA-SF, Mini-Nutritional Assessment Short-Form; SPPB, Short Physical Performance Battery; BNP, B-type Natriuretic Peptide; LVEF, Left Ventricular Ejection Fraction.

## 4 Discussion

Elderly patients, characterized by diminished physiological reserve, increased vulnerability, and reduced homeostatic capacity (24), constitute a high-risk demographic for developing HF and frailty. A plethora of preceding studies has documented a prevalence of frailty ranging from 15% to 74% in patients with HF (25). In the present research, 298 hospitalized elderly patients with HF were enrolled, and the prevalence of coexisting frailty was found to be 78.5%, which lies at the upper limit of previously reported ranges. This finding may be related to the high proportion of very old patients and those with prolonged or recurrent hospitalizations. These results, together with previous findings (26), confirm the high coexistence rate of HF and frailty within the elderly demographic, thereby underscoring the necessity for meticulous clinical observation.

Stratified analyses by frailty severity showed that greater frailty severity was associated with older age, higher comorbidity burden, lower body weight, BMI, dominant calf circumference, ADL, IADL, MNA-SF, SPPB, POMA, and grip strength (all *P* < 0.05). No inter-group differences were observed for sex, hypertension, coronary artery disease, arrhythmia, or electrocardiographic parameter (all *P* > 0.05). The absence of a sex difference is consistent with some reports but not others (27, 28), possibly because of differences in inclusion criteria and age composition across studies.

Our stratified analyses showed that frailty severity was correlated with multiple demographic, functional, and laboratory-derived parameters. Demographically, older age and higher comorbidity burden were associated with greater frailty status. Frailty status was also associated with markers of cardiac dysfunction (higher BNP, lower LVEF), malnutrition-related indices (lower BMI, dominant calf circumference, and MNA-SF scores), and impaired physical performance (lower ADL, IADL, SPPB, and POMA scores). Previous studies have also emphasized the clinical relevance of malnutrition and impaired physical performance in HF populations (29, 30). Prior publications have proposed several pathways linking HF to malnutrition and physical impairment, including gastrointestinal and splanchnic congestion (31), as well as diuretic-related disturbances, chronic inflammation, and neuroendocrine activation (32). However, these mechanistic hypotheses cannot be tested using the present cross-sectional study.

Also, the present study revealed that serum BNP levels differed across frailty groups, and significant intergroup differences were observed in reduced ejection fraction, but BNP was not significantly associated with frailty severity after multivariable adjustment in the PPOM. This may be related to the characteristics of our hospitalized elderly study population, that is, the effect of BNP may have been masked by other clinical variables after multivariable adjustment. Notably, continuous LVEF was significantly lower in the severe frailty group than in the non-frailty group, while the proportion of reduced ejection fraction was higher in the severe frailty group than in both other groups (*P* < 0.05). In the categorical primary PPOM, LVEF >50% was associated with lower odds of being in a higher frailty category (OR = 0.46, 95% CI: 0.21–0.99; *P* = 0.048). Moreover, the association with LVEF in the categorical analysis was not reproduced when LVEF was entered continuously in the sensitivity analysis, suggesting that this result may be sensitive to variable specification and should be interpreted cautiously. These findings are partly consistent with previous studies reporting associations between cardiac dysfunction and frailty (33, 34). Previous studies (35–39) have indicated that frailty overlaps with heart failure in clinical evolution and is associated with adverse outcomes and long-term functional deterioration. Moreover, several common pathophysiological pathways, involving inflammation, neuroendocrine activation, metabolism, nutrition, and physical function, may jointly contribute to these associations (40–42), but these mechanisms were not directly examined in the present study.

The multivariable PPOM analysis showed that the presence of multiple comorbidities, lower MNA-SF, SPPB and LVEF levels were important factors associated with frailty in elderly HF patients. These findings are broadly consistent with previous reports (43, 44). Of note, our study found that age and an MNA-SF score of 8–11 (indicating risk of malnutrition) were statistically significant in discriminating non-frailty from frailty, but showed no significant difference in comparisons within frailty severity levels. One possible explanation is that patients with severe frailty not only exhibit physiological age-related changes and mild nutritional risk, but more often present with a combination of multiple factors, including heavy comorbidity burden, impaired physical function, and decreased cardiac function, leading to a decline in multi-system reserve capacity. Consequently, the association between age or mild nutritional abnormalities and frailty may be partially masked or attenuated by the complex interplay of other factors. Although the specific indicators varied across studies, all have consistently indicated that multiple pathological burdens, cardiac dysfunction, poor nutritional status, and physical impairment are key factors associated with frailty in elderly HF patients. Overall, these findings provide observational support for considering comorbidity burden, nutritional status, physical performance, and cardiac function when assessing frailty in hospitalized older patients with HF.

The present study has several limitations that should be acknowledged. (1) This was a single-center, cross-sectional study with a relatively limited sample size, which may have introduced selection bias and limits the generalizability of our findings to other centers, geographic regions, or healthcare systems. (2) Retrospective data collection precluded systematic incorporation of information on HF phenotype, clinical presentation/severity, etiology, and treatment, limiting comprehensive characterization of the HF population. (3) Frailty was assessed only once during hospitalization using the CFS, precluding causal or temporal inferences and the capture of dynamic changes. (4) The exclusion of patients with CFS scores of 8–9 may have led to an underestimation of the true prevalence of severe frailty, although the observed prevalence in our cohort (78.5%) remained at the upper end of previously reported ranges (15%–74%). (5) The self-constructed ECG and Echo abnormality scores lacked external validation and were used only for baseline comparisons. (6) The LSD t-test did not adjust for multiple comparisons. The pairwise results should be interpreted with caution. (7) Wide confidence intervals for some threshold-specific estimates and changes in significance when categorical variables were entered continuously suggest limited statistical power due to sample size constraints, warranting cautious interpretation and external validation. (8) The CFS relies on clinical judgment and may be subject to assessor variability, although all assessments were performed by trained geriatric nurses using standardized protocols. Future multicenter, large-sample, prospective studies with longitudinal designs are warranted to validate these findings and to explore the bidirectional causal pathways linking heart failure and frailty in elderly patients.

Despite these limitations, this study has several strengths. First, it focuses on hospitalized elderly heart-failure patients with a high median age of 83 years, an under-investigated vulnerable real-world subgroup. Second, we included a moderate sample of 298 participants covering a broad age spectrum (60–101 years) with balanced sex distribution. Third, frailty severity was graded using the Clinical Frailty Scale, alongside multidimensional assessments of demographic, nutritional, physical-performance, and cardiac indices. Finally, the present cross-sectional analysis identified clinically relevant correlates of frailty severity, including comorbidity burden, nutritional status, physical performance, and cardiac function, which may support risk-stratified frailty assessment in hospitalized older HF patients.

## 5 Conclusion

In summary, hospitalized elderly HF patients show a high prevalence of frailty. Comorbidities, poor nutritional status (lower MNA-SF score), impaired physical function (lower SPPB score), and reduced LVEF are important correlates of frailty severity in these patients.

## Data Availability

The original contributions presented in the study are included in the article/Supplementary Material, further inquiries can be directed to the corresponding author/s.
